# Neurocutaneous Syndromes, Perinatal Factors, and the Risk of Childhood Cancer in Sweden

**DOI:** 10.1001/jamanetworkopen.2023.25482

**Published:** 2023-07-25

**Authors:** Christina-Evmorfia Kampitsi, Ann Nordgren, Hanna Mogensen, Emeli Pontén, Maria Feychting, Giorgio Tettamanti

**Affiliations:** 1 Unit of Epidemiology, Institute of Environmental Medicine, Karolinska Institutet, Stockholm, Sweden; 2 Department of Molecular Medicine and Surgery, Karolinska Institutet, Stockholm, Sweden; 3 Department of Clinical Genetics, Karolinska University Hospital, Stockholm, Sweden

## Abstract

**Importance:**

Neurocutaneous syndromes are associated with cancer predisposition and sometimes associated with perinatal factors. A better understanding of the association between neurocutaneous syndromes, perinatal factors, and childhood cancer is key for earlier cancer detection.

**Objective:**

To evaluate the association of neurocutaneous syndromes and perinatal factors with childhood cancer risk in a cohort of Swedish children.

**Design, Setting, and Participants:**

In this nationwide cohort study, all children and adolescents up to age 20 years, from 1973 to 2015, were identified through the Swedish National Medical Birth Register (MBR), provided they had information on both biological parents. Analyses were conducted from April 2021 through May 2023.

**Exposures:**

Diagnoses of neurocutaneous syndromes were obtained from the MBR, National Patient Register, and Cause of Death register. Perinatal factors (birth weight, gestational age, birth weight by gestational age, 5-minute Apgar score, and head circumference) were obtained from the MBR.

**Main Outcomes and Measures:**

Childhood cancer risk (<20 years at diagnosis; identified from the National Cancer Register), including leukemia, lymphoma, and central nervous system (CNS) tumors.

**Results:**

Among 4 173 108 included children (2 143 133 [51.4%] male, median [IQR] follow-up 20 [9.7-20] years), 1783 had neurofibromatosis type 1 (NF1), 444 tuberous sclerosis, 63 von Hippel-Lindau disease, and 39 ataxia-telangiectasia. An increased cancer risk was observed among children with any neurocutaneous syndrome (HR, 34.9; 95% CI, 30.8-39.6) and was particularly pronounced for CNS tumors (HR, 111.7; 95% CI, 96.8-128.8), except among children with ataxia-telangiectasia, where the increased risk was associated with lymphomas (HR, 233.1; 95% CI, 75.0-724.1). Leukemia risk was increased only among children with NF1 (HR, 4.1; 95% CI, 1.7-9.8). Several perinatal factors, including high birth weight, being born large for gestational age, preterm birth, low 5-minute Apgar score, and large head circumference had lesser associations with childhood cancer. Adjusting for neurocutaneous syndromes did not affect these associations.

**Conclusions and Relevance:**

In this nationwide cohort study, neurocutaneous syndromes were associated with an increased risk of childhood cancer, especially CNS tumors. Several perinatal factors had lesser associations with childhood cancer, independently of the presence of neurocutaneous syndromes. Other biological mechanisms likely underlie the association between perinatal factors and childhood cancer.

## Key Points

**Question:**

How are neurocutaneous syndromes and perinatal factors associated with childhood cancer?

**Findings:**

In this cohort study of more than 4 million children born in Sweden between 1973 and 2014, children with any neurocutaneous syndrome had a markedly increased cancer risk, particularly pronounced for central nervous system and brain tumors. Lesser associations between perinatal factors and childhood cancer were not explained by neurocutaneous syndromes.

**Meaning:**

In this study. neurocutaneous syndromes were associated with a considerably increased risk of childhood cancer; the association between perinatal factors and childhood cancer, however, appeared to originate from other biological mechanisms not involving neurocutaneous syndromes.

## Teaser

This cohort study evaluates the association between neurocutaneous syndromes, perinatal factors, and the risk of childhood cancer in Sweden.

## Introduction

Neurocutaneous syndromes, or phakomatoses, arise at conception and are a phenotypically and genetically diverse group of multisystem congenital syndromes which, in addition to cutaneous, ocular, and neural manifestations, are associated with an increased risk of tumor development, particularly within the central and peripheral nervous systems.^1^ These cancer-predisposing syndromes include disorders such as neurofibromatosis type 1 (NF1) and type 2 (NF2), tuberous sclerosis complex (TSC), Sturge-Weber syndrome, von Hippel-Lindau disease, and ataxia-telangiectasia.^1^ The most common neurocutaneous syndromes are NF1 and TSC, with an estimated prevalence at birth as high as 1 in 2000 and 1 in 6000, respectively.^1,2^

Although the cause of most childhood cancers remains largely unknown, established risk factors for childhood cancer other than cancer-predisposing syndromes, such as neurocutaneous syndromes, are high-dose ionizing radiation, prior chemotherapy, age, sex, and race and ethnicity.^3^ Several perinatal factors have also been associated with different childhood cancers; these factors include preterm birth, high birth weight, low Apgar score, large head circumference, and advanced parental age.^4,5,6,7,8,9^

Neurocutaneous syndromes may also, depending on diagnosis, be associated with some of these perinatal factors. Recent studies have highlighted associations between advanced parental age and de novo neurocutaneous syndromes in the offspring, and higher birth weight, larger head circumference, and slightly shorter gestational length have been observed in individuals born with NF1.^10,11,12,13^ Neurocutaneous syndromes and perinatal factors, therefore, are associated with each other and with the risk of childhood cancer. However, they have seldom been studied simultaneously, and there is currently little knowledge about the role neurocutaneous syndromes play in the associations between perinatal factors and childhood cancer risk. Elucidating these associations is of value both for the understanding of childhood cancer causes and for identifying children at increased cancer risk. Thus, our aim was to quantify the risk of cancer in children with different neurocutaneous syndromes and to assess to what extent the previously reported associations between perinatal factors and childhood cancer could be explained by neurocutaneous syndromes.

## Methods

This nationwide, population-based cohort study was approved by the regional ethical review board in Stockholm, Sweden. Informed consent was waived, as the study was register-based. This study adhered to the Strengthening the Reporting of Observational Studies in Epidemiology (STROBE) reporting guideline.

In this study, all children born 1973 to 2014 and registered in the Swedish National Medical Birth Register (MBR) with information on both biological parents were eligible for inclusion (eFigure 1 in Supplement 1). The MBR was established in 1973 and comprises data on nearly all deliveries in Sweden.^14^ Cancer diagnoses were retrieved by linking the MBR to the National Cancer Register through the unique personal identity number.^15^ The National Cancer Register, established in 1958, covers the entire population; reporting of all newly detected cancers is mandatory and completeness is high (around 96%).^16^ Children enrolled in the study were followed from birth until the first of the following events: cancer diagnosis, death, emigration, 20th birthday, or end of follow-up (end of December 2015). Information on emigrations was obtained from the Total Population Register,^17^ whereas date of death was retrieved from the National Cause of Death Register.^18^

Individuals were defined as having a neurocutaneous syndrome if they had been assigned at least 1 diagnostic code for phakomatoses in the MBR, the National Patient Register,^19^ or the Cause of Death Register. The National Patient Register was initiated in 1964 and became nationwide in 1987. It includes all hospitalizations; outpatient specialist care is also included from 2001. By combining these registers, we were able to retrieve lifelong information on neurocutaneous syndrome diagnoses for the study population. The neurocutaneous syndromes we examined separately were NF1, TSC, von Hippel-Lindau disease, and ataxia-telangiectasia. We did not examine Sturge-Weber syndrome separately, as no children with Sturge-Weber syndrome developed cancer during follow-up. The Swedish *International Classification of Diseases, *Revisions 8 through 10 (*ICD-8*,* ICD-9*, and* ICD-10*) codes used in the registers do not differentiate between NF1 and NF2, therefore we identified probable NF1 diagnoses (denoted as NF1 from here on) by excluding individuals with a neurofibromatosis diagnosis who also developed a benign meningioma or acoustic neuroma, tumors that typically occur in individuals with NF2. As no specific diagnostic code for von Hippel-Lindau disease is available in *ICD-9*, children who both were born and died between 1987 and 1996, when *ICD-9* was used, were excluded from the corresponding analyses. With regard to the outcome of interest, incident cancer was defined as the first diagnosis of primary cancer in the Cancer Register, categorized by the authors according to the *International Classification of Childhood Cancer* (third version).^20^ Benign tumors other than central nervous system (CNS) tumors were excluded from this study.

Perinatal factors were obtained from the MBR, and include birth weight (categorized as low [<2500 g], normal [2500-4000 g], and high [>4000 g]), gestational age (categorized as preterm [<37 weeks], full term [37-42 weeks], and postterm [>42 weeks]), birth weight by gestational age (categorized as small, appropriate, and large, using the 10th and 90th percentiles of the birth weight distribution in the population, according to sex, birth decade, and gestational age), 5-minute Apgar score (categorized as very low [0-3], low [4-6], and reassuring [7-10]), and head circumference (categorized as <33 cm, 35 cm, 36-37 cm, and >37 cm).

### Statistical Analysis

First, we evaluated the association between different neurocutaneous syndromes and childhood cancer risk, including specific subtypes, by using Cox proportional hazards regression models to estimate hazard ratios (HR) and corresponding 95% CIs. In this analysis, children with each neurocutaneous syndrome were compared with children without. Subsequently, similar analyses were performed to assess the association between perinatal factors and the risk of childhood cancer. We then adjusted the latter analyses for the presence of any neurocutaneous syndrome to determine whether the associations between perinatal factors and childhood cancer risk could be confounded by neurocutaneous syndromes. To assess the proportional hazards assumption, we used the Schoenfeld residuals test; where violations were detected, we conducted additional analyses stratified by age at cancer diagnosis.

We further performed several sensitivity analyses to challenge our findings; first, to gauge the possibility of an interaction between neurocutaneous syndromes and certain perinatal factors on the risk of childhood cancer. Analyses stratified by study period (children born 1973-1995 or 1996-2015) were also performed to evaluate the possibility of differential exposure misclassification, as outpatient care is not covered during the early study period. In addition, we conducted an analysis considering only children with a familial neurocutaneous syndrome (ie, children with a neurocutaneous syndrome and at least 1 first or second degree relative or cousin with a diagnosis of a neurocutaneous syndrome). Thereby, we ensured that a neurocutaneous syndrome diagnosis was not likely obtained only due to the increased medical monitoring in relation to a cancer diagnosis. A final sensitivity analysis considered only children with at least 2 diagnoses of a neurocutaneous syndrome. All analyses were adjusted for sex, birth year (1973-1979, 1980-1989, 1990-1999, 2000-2009, or 2010-2015), parental age (≤25, 26-29, 30-34, 35-39, or ≥40 years), parental education (primary, secondary, or postsecondary), and health care region of residence at birth (6 regions) (eFigure 2 in Supplement 1). This information was retrieved from the MBR, the national censuses, and the longitudinal integrated database for health insurance and labor market studies.^21^ Individuals with known childhood cancer predisposition syndromes other than neurocutaneous syndromes^22,23^ or missing information on the main covariates were excluded from the analysis (eFigure 1 and eTable 1 in Supplement 1). Data were prepared with SAS version 9.4 (SAS Institute), whereas all analyses were performed with Stata version 14.2 (StataCorp). The significance threshold was 2-tailed *P* = .05. Data were analyzed from April 2021 to May 2023.

## Results

This study included 4 173 108 children and adolescents aged 20 years or younger (2 143 133 [51.4%] male), followed over a median (IQR) of 20 (9.7-20) years. Among them, we identified 2791 individuals with neurocutaneous syndrome diagnoses. Of those, 1902 had neurofibromatosis (including 1783 NF1, as described in the Methods), 444 TSC, 63 von Hippel-Lindau disease, and 39 had ataxia-telangiectasia (median [IQR] follow-up among children with neurocutaneous syndromes, 19.9 [10.7-20] years). Birth and parental characteristics of the participants are reported in Table 1. Participants with neurocutaneous syndrome diagnoses were predominantly male, with a higher proportion born to older fathers (≥40 years) and a lower proportion born to parents with postsecondary education compared with the neurocutaneous syndrome–free population.

**Table 1.** Baseline Characteristics of the Study Cohort by Neurocutaneous Syndrome Status^a^

Characteristic	No. (%)	
	Neurocutaneous syndrome–free	Neurocutaneous syndromes
Sex		
Male	2 143 133 (51.4)	1469 (52.6)
Female	2 027 204 (48.6)	1322 (47.4)
Maternal age, y		
≤25	1 202 358 (28.8)	781 (28.0)
26-29	1 184 145 (28.4)	822 (29.5)
30-34	1 185 312 (28.4)	779 (27.9)
35-39	501 561 (12.1)	336 (12.0)
≥40	96 941 (2.3)	73 (2.6)
Paternal age, y		
≤25	637 949 (15.3)	403 (14.4)
26-29	1 024 479 (24.5)	643 (23.0)
30-34	1 328 592 (31.9)	875 (31.4)
35-39	759 726 (18.2)	521 (18.7)
≥40	419 571 (10.1)	349 (12.5)
Maternal education		
Primary	524 832 (12.6)	435 (15.6)
Secondary	1 903 375 (45.6)	1363 (48.8)
Postsecondary	1 742 110 (41.8)	993 (35.6)
Paternal education		
Primary	772 426 (18.5)	631 (22.6)
Secondary	2 024 695 (48.6)	1390 (49.8)
Postsecondary	1 373 196 (32.9)	770 (27.6)

^a^ *International Classification of Diseases* (*ICD*) codes defined as neurocutaneous syndromes: 74340, 75960, 75982, 75986, 75987 (*ICD-8*); 237H, 334W, 759F, 759G (*ICD-9*); G113, Q850, Q851, Q858, Q858A, Q858B, Q858C, Q859 (*ICD-10*). The conditions described by these diagnostic codes include neurofibromatosis, tuberous sclerosis complex, Sturge-Weber syndrome, von Hippel-Lindau disease, ataxia-telangiectasia, Peutz-Jeghers syndrome, other specified phakomatoses, and other unspecified phakomatoses.

Among the study population, we identified 10 759 children with cancer, including 2896 CNS tumors, 2849 leukemias, and 1273 lymphomas. Almost 9% of individuals (247 participants) with neurocutaneous syndromes had a childhood cancer diagnosis, compared with about 0.22% in the neurocutaneous syndrome-free population (10 512 individuals); the cumulative incidence of cancer among children with and without a neurocutaneous syndrome is presented in eFigure 3 in Supplement 1. The overall adjusted HR for all cancers in children with any neurocutaneous syndrome, compared with the general population, was 34.9 (95% CI, 30.8-39.6) (Table 2 and eTable 2 in Supplement 1). The adjusted HR varied from 22.4 (95% CI, 8.4-59.7) among individuals with von Hippel-Lindau disease to 31.3 (95% CI, 26.5-36.9) in NF1. The increased risk was associated with CNS tumors in every neurocutaneous syndrome (HR for any neurocutaneous syndrome, 111.7; 95% CI, 96.8-128.8) except ataxia-telangiectasia, where the increased cancer risk was associated with lymphoma (HR, 233.1; 95% CI, 75.0-724.1), but the study population included only 3 lymphoma cases with ataxia-telangiectasia. The risk of CNS tumors was more pronounced in children with TSC (adjusted HR [aHR], 99.4; 95% CI, 69.8-141.7). Similarly increased risk was observed for malignant CNS tumors (eTable 3 in Supplement 1). There was also an increased risk of childhood leukemias among individuals with NF1 (aHR, 4.1; 95% CI, 1.7-9.8). Results were similar in separate analyses of children born up to and after 1995 (eTable 4 in Supplement 1) and in children born with familial neurocutaneous syndromes (eTable 5 in Supplement 1). In the latter analysis, the association between NF1 and CNS tumors was somewhat attenuated, whereas even greater associations were observed for other neurocutaneous syndromes. Somewhat greater associations were observed when defining only children with at least 2 diagnoses of a neurocutaneous syndrome as exposed (eTable 6 in Supplement 1). Results stratified by age at cancer diagnosis are presented in eTable 7 in Supplement 1.

**Table 2.** Risk of Cancer Among Children Born 1973 to 2014 With Neurocutaneous Syndromes in Sweden^a^

Cancer type	NS		NF1		TSC		VHL^b^		AT	
	No. of cases, NS/no NS	HR (95% CI)	No. of cases, NF1/no NF1	HR (95% CI)	No. of cases, TSC/no TSC	HR (95% CI)	No. of cases, VHL/no VHL	HR (95% CI)	No. of cases, AT/no AT	HR (95% CI)
All cancers	247/10 512	34.9 (30.8-39.6)	142/10 617	31.3 (26.5-36.9)	36/10 723	31.0 (22.4-43.0)	4/10 575	22.4 (8.4-59.7)	3/10 756	27.4 (8.8-84.9)
CNS	203/2693	111.7 (96.8-128.8)^c^	112/2784	93.8 (77.6-113.3)^b^	31/2865	99.4 (69.8-141.7)^b^	<3/2837	41.0 (10.3-164.2)^c^	NA	NA
Leukemia	5/2844	2.6 (1.1-6.2)^c^	5/2844	4.1 (1.7-9.8)^b^	NA	NA	NA	NA	NA	NA
Lymphoma	4/1269	4.7 (1.8-12.5)^c^	NA	NA	NA	NA	NA	NA	3/1270	233.1 (75.0-724.1)^c^

Abbreviations: AT, ataxia-telangiectasia; CNS, central nervous system; HR, hazard ratio; NA, not applicable; NF1, neurofibromatosis 1; NS, neurocutaneous syndromes; TSC, tuberous sclerosis complex; VHL, von Hippel-Lindau.

^a^ Adjusted for sex, birth decade, maternal/paternal age, maternal/paternal education, and region of residence at birth.

^b^ Excluding children who were both born and died between 1987 and 1996.

^c^ Not meeting proportional hazards assumption.

The association between perinatal factors and childhood cancer is shown in Table 3 (unadjusted estimates in eTable 8 in Supplement 1). The adjusted HR of all cancers in children with a high birth weight was 1.2 (95% CI, 1.1-1.2), most pronounced for lymphomas (aHR, 1.3; 95% CI, 1.1-1.4). Similar results were observed for children born large for gestational age. Children born with large head circumferences had a marginally higher risk of leukemia and lymphoma. After additionally adjusting the analyses for diagnosis of any neurocutaneous syndrome, the associations between perinatal factors and cancer risks remained virtually unchanged. However, among children with NF1, the association between being born large for gestational age or with a low 5-minute Apgar score, and childhood cancer was slightly greater (eTable 9 in Supplement 1). Moreover, we found indications of a higher risk estimate among children who both had NF1 and were born preterm, large for gestational age, or with a large head circumference (eTable 10 in Supplement 1).

**Table 3.** Risk of Cancer Among Children Born With Different Perinatal Factors 1973 to 2014 in Sweden

Perinatal factor	All cancers (n = 10 759)			CNS (n = 2896)			Leukemia (n = 2849)			Lymphoma (n = 1273)		
	No. cases	Model 1^a^	Model 2^b^	No. cases	Model 1^a^	Model 2^b^	No. cases	Model 1^a^	Model 2^b^	No. cases	Model 1^a^	Model 2^b^
Birth weight category												
Low	448	1.1 (1.0-1.2)	1.1 (1.0-1.2)	115	1.0 (0.8-1.2)^c^	1.0 (0.8-1.2)^c^	121	1.1 (0.9-1.3)^c^	1.1 (0.9-1.3)^c^	47	1.0 (0.7-1.3)^c^	1.0 (0.7-1.3)^c^
Normal	8167	1 [Reference]	1 [Reference]	2233	1 [Reference]	1 [Reference]	2141	1 [Reference]	1 [Reference]	949	1 [Reference]	1 [Reference]
High	2101	1.2 (1.1-1.2)	1.2 (1.1-1.2)	531	1.1 (1.0-1.2)^c^	1.1 (1.0-1.2)^c^	577	1.2 (1.1-1.3)^c^	1.2 (1.1-1.3)^c^	272	1.3 (1.1-1.4)^c^	1.2 (1.1-1.4)^c^
Birth weight for gestational age												
SGA	1038	1.0 (0.9-1.0)	1.0 (0.9-1.0)	277	1.0 (0.8-1.1)^c^	1.0 (0.8-1.1)^c^	291	1.0 (0.9-1.2)^c^	1.0 (0.9-1.2)^c^	123	1.0 (0.8-1.2)^c^	1.0 (0.8-1.2)^c^
AGA	8401	1 [Reference]	1 [Reference]	2285	1 [Reference]	1 [Reference]	2220	1 [Reference]	1 [Reference]	979	1 [Reference]	1 [Reference]
LGA	1289	1.2 (1.1-1.3)	1.2 (1.1-1.2)	323	1.1 (1.0-1.2)^c^	1.1 (0.9-1.2)^c^	334	1.2 (1.0-1.3)^c^	1.2 (1.0-1.3)^c^	166	1.3 (1.1-1.6)^c^	1.3 (1.1-1.6)^c^
5-min Apgar score												
0-3	31	1.4 (1.0-1.9)	1.4 (1.0-1.9)	7	1.1 (0.5-2.4)^c^	1.1 (0.5-2.4)^c^	5	0.8 (0.3-2.0)^c^	0.8 (0.3-2.0)^c^	3	1.0 (0.3-3.2)^c^	1.0 (0.3-3.2)^c^
4-6	98	1.2 (1.0-1.5)	1.2 (1.0-1.5)	24	1.1 (0.8-1.7)^c^	1.1 (0.7-1.6)^c^	26	1.2 (0.8-1.8)^c^	1.2 (0.8-1.8)^c^	<3	0.2 (0.1-0.8)^c^	0.2 (0.1-0.8)^c^
7-10	9867	1 [Reference]	1 [Reference]	2627	1 [Reference]	1 [Reference]	2629	1 [Reference]	1 [Reference]	1191	1 [Reference]	1 [Reference]
Head circumference												
<33 cm	804	1.1 (1.0-1.2)	1.1 (1.0-1.2)	202	0.9 (0.8-1.0)^c^	0.9 (0.8-1.0)^c^	212	1.1 (1.0-1.3)^c^	1.1 (1.0-1.3)^c^	106	1.3 (1.0-1.6)^c^	1.3 (1.0-1.6)^c^
33-34 cm	3458	1 [Reference]	1 [Reference]	1023	1 [Reference]	1 [Reference]	867	1 [Reference]	1 [Reference]	375	1 [Reference]	1 [Reference]
35 cm	2640	1.0 (1.0-1.1)	1.0 (1.0-1.1)	677	0.9 (0.8-1.0)^c^	0.9 (0.8-1.0)^c^	745	1.1 (1.0-1.3)^c^	1.1 (1.0-1.3)^c^	303	1.1 (0.9-1.3)^c^	1.1 (0.9-1.3)^c^
36-37 cm	3070	1.1 (1.0-1.2)	1.1 (1.0-1.2)	790	1.0 (0.9-1.1)^c^	1.0 (0.9-1.1)^c^	828	1.1 (1.0-1.3)^c^	1.1 (1.0-1.3)^c^	407	1.3 (1.1-1.5)^c^	1.3 (1.1-1.5)^c^
>37 cm	415	1.3 (1.1-1.5)	1.3 (1.1-1.4)	106	1.1 (0.9-1.4)^c^	1.1 (0.9-1.4)^c^	110	1.3 (1.0-1.6)^c^	1.3 (1.0-1.6)^c^	50	1.3 (1.0-1.8)^c^	1.3 (1.0-1.8)^c^
Preterm birth	649	1.1 (1.1-1.2)	1.1 (1.0-1.2)	168	1.1 (0.9-1.2)^c^	1.0 (0.9-1.2)^c^	163	1.0 (0.9-1.2)^c^	1.0 (0.9-1.2)^c^	69	1.0 (0.8-1.2)^c^	1.0 (0.8-1.2)^c^

Abbreviations: AGA, appropriate for gestational age; CNS, central nervous system; LGA, large for gestational age; SGA, small for gestational age.

^a^ Adjusted for sex, birth decade, maternal/paternal age, maternal/paternal education, region of residence at birth.

^b^ Additionally adjusted for any neurocutaneous syndrome diagnosis.

^c^ Not meeting proportional hazards assumption.

Finally, when assessing the risk of childhood cancer in the first 5 years of life, higher risk estimates were observed, especially for very low 5-minute Apgar scores (aHR, 2.0; 95% CI, 1.2-3.2) (eTable 11 in Supplement 1). The association was even more pronounced among children diagnosed with cancer in the first 2 years of life (aHR for all cancers, 3.2; 95% CI, 2.0-5.6; aHR for CNS tumors, 3.4; 95% CI, 1.1-10.7). The risk estimates for large head circumference and preterm birth followed a similar pattern. As with the main analyses, no attenuations were observed after adjusting for neurocutaneous syndromes.

## Discussion

In this national cohort study of more than 4 million children, we found an increased cancer risk in children with any neurocutaneous syndrome, which was associated with CNS tumors, except for children with ataxia-telangiectasia, who had a markedly elevated risk of lymphoma. A modestly increased leukemia risk was observed only among children with NF1. We also identified several perinatal factors (high birth weight, large for gestational age, preterm birth, low 5-minute Apgar score, and large head circumference) that were associated with childhood cancer, although these were often lesser associations. Yet, we observed greater associations between certain perinatal factors and early childhood cancer risk. Moreover, regardless of the greater association between neurocutaneous syndromes and childhood cancer risk, adjusting for the former did not explain the observed associations between perinatal factors and cancer risk. Finally, those born with both NF1 and the perinatal factors of interest had indications of a higher risk of childhood cancer.

Although the cause of most childhood cancers remains largely unknown, it has long been recognized that, in a subset of children, tumors develop in the setting of underlying, often inherited neurocutaneous syndromes.^24^ In alignment with our findings, NF1, TSC, and von Hippel-Lindau disease have been consistently associated with increased cancer risk, especially CNS tumors, in both children and adults^25,26^; individuals with ataxia-telangiectasia are considered to have an increased predisposition for cancers of lymphoid origin predominantly.^27^ However, the associations reported in the literature mostly stem from case series conducted in specialized referral centers; prospective, large-scale studies are especially sparse and mainly conducted among individuals with NF1. Although previous cohort studies on NF1 and cancer risk reported lesser associations than what we have observed in our study, they did not focus on pediatric cancer, but instead investigated cancer risk at any age^28^ and in individuals younger than 50 years.^29^ To date, only 1 Finnish study^30^ has evaluated the risk of pediatric cancer among children with NF1, with findings similar to ours.

Several perinatal factors have also been described as potential factors associated with risk for childhood cancer. In agreement with our findings, a long series of population-based studies in Sweden have highlighted that infants born large for gestational age are at increased risk of brain tumors, Hodgkin and non-Hodgkin lymphoma, and leukemia.^4,5,6,7,8^ A nationwide, register-based cohort study in Denmark and Sweden proposed a low 5-minute Apgar score as a predictor of childhood cancer, especially for cancer diagnosed before 6 months of age.^31^ In a case-control study based on the Danish, Finnish, Norwegian, and Swedish health registries, high birth weight, being born small or large for gestational age, preterm birth, large head circumference, and low 5-minute Apgar score were associated with an increased risk of childhood CNS tumors.^9^

Yet, the association of some perinatal factors with childhood cancer risk has been conflicting: a recent systematic review and meta-analysis on cancer risk in children and young adults born preterm found solely an increased hepatoblastoma risk.^32^ As these associations are often also lesser, the question arises whether they could be explained by underlying conditions, predisposing to both certain perinatal factors and childhood cancer. Today, more than 150 rare childhood cancer predisposition syndromes are known and several among them affect perinatal factors. However, although the association between neurocutaneous syndromes and childhood cancer seems to be great, the former did not explain the associations observed between specific perinatal factors and childhood cancer in this study, likely because of their low prevalence in the population. Although other genetic syndromes might partially explain the effect of perinatal factors on childhood cancer, it is important to recognize that additional biological mechanisms are likely involved. First, the neonates with poor birth outcomes could have been exposed to potentially cytotoxic agents because of highly intensive neonatal care.^9^ Second, high birth weight and increased fetal growth are related to more cells potentially at risk for carcinogenesis, as well as potentially higher intrauterine cell proliferation.^9^ Finally, reverse causality could also play a role, as intrauterine tumors might affect how the fetus grows.^9^

### Strengths and Limitations

To our knowledge, this population-based study is the largest to have examined the effect of various neurocutaneous syndromes on childhood cancer risk, as well as the first to have evaluated whether the reported associations between perinatal factors and childhood cancer could partly be attributed to neurocutaneous syndromes. The register-based design allowed us to obtain information on perinatal factors as well as diagnoses of neurocutaneous syndromes and cancer for over 4 million children born in Sweden in a period of over 40 years. One limitation of the current study is that while the National Patient Register was initially founded in 1964, it did not achieve universal coverage of inpatient care until 1987—and outpatient care coverage begun in 2001. Consequently, we might have missed some neurocutaneous syndromes diagnosed in the early study period. To assess the possibility that hospitalization for a cancer diagnosis could have led to a higher likelihood that a neurocutaneous syndrome diagnosis was registered, we conducted separate analyses for children born up to and after 1995. Results for the 2 periods were similar, rendering such an eventuality less likely. However, the possibility that some mild neurocutaneous syndromes might have been diagnosed only due to the increased medical monitoring in relation to a cancer diagnosis cannot be excluded; thus, we might have overestimated the association between neurocutaneous syndromes and childhood cancer. Yet, results from the sensitivity analysis where we examined the childhood cancer risk associated with familial neurocutaneous syndromes do not support this hypothesis. Moreover, due to the Swedish *ICD*-coding system, we could only distinguish NF1 from NF2 based on the absence of benign meningioma or acoustic neuroma diagnoses. However, it is unlikely that the few NF2 individuals that might have been misclassified as NF1 have impacted the results, as NF2 overall constitutes only a fraction of all patients with neurofibromatosis. Additionally, the low prevalence of neurocutaneous syndromes in the population resulted in high uncertainty around some of the reported estimates of the effect of individual neurocutaneous syndromes on specific cancer types.

## Conclusions

In this nationwide cohort study, certain neurocutaneous syndromes were associated with an increased childhood cancer risk, especially CNS tumors. Specific perinatal factors, such as high birth weight, large for gestational age, preterm birth, low 5-minute Apgar score, and large head circumference, had lesser associations with childhood cancer; greater associations were observed with a diagnosis of childhood cancer at a young age. However, none of these associations were explained by neurocutaneous syndromes; therefore, other biological mechanisms likely underlie any association between specific perinatal factors and childhood cancer. Our findings underscore the importance of the recently published cancer screening recommendations in children with NF1^33^ and TSC.^34^

## Notes

Supplement 1. **eFigure 1. **Cohort Flow Chart **eFigure 2. **Directed Acyclic Graph of the Study **eFigure 3. **Cumulative Incidence of Cancer Among Children With and Without a Neurocutaneous Syndrome Diagnosis **eTable 1. **Distribution of Exposure and Main Outcomes in the Study Population, According to Completeness of Data on the Main Covariates **eTable 2. **Risk of Cancer Among Children Born 1973 to 2014 With Neurocutaneous Syndromes in Sweden (Unadjusted Hazard Ratios and 95% CIs) **eTable 3. **Risk of Malignant CNS Among Children Born 1973 to 2014 With Neurocutaneous Syndromes in Sweden (Hazard Ratios and 95% CIs) **eTable 4. **Risk of Cancer Among Children Born 1973 to 2014 With Neurocutaneous Syndromes in Sweden, Stratified by Birth Period (Hazard Ratios and 95% CIs) **eTable 5. **Risk of Cancer Among Children Born 1973 to 2014 With Familial Neurocutaneous Syndromes in Sweden (Hazard Ratios and 95% CIs) **eTable 6. **Risk of Cancer Among Children Born 1973 to 2014 With Neurocutaneous Syndromes in Sweden (at Least 2 Diagnoses of a Neurocutaneous Syndrome; Hazard Ratios and 95% CIs) **eTable 7. **Risk of Cancer Among Children Born 1973 to 2014 With Neurocutaneous Syndromes in Sweden, Stratified by Age at Cancer Diagnosis (Ratios and 95% CIs) **eTable 8. **Risk of Cancer Among Children Born With Different Perinatal Factors 1973 to 2014 in Sweden (Unadjusted Hazard Ratios and 95% CIs) **eTable 9. **Risk of Cancer Among Children Born With Different Perinatal Factors 1973 to 2014 in Sweden, Stratified by Neurofibromatosis Type 1 Diagnosis (Hazard Ratios and 95% CIs) **eTable 10. **Risk of Cancer Among Children Born 1973 to 2014 With Different Combinations of Neurofibromatosis Type 1 and Specific Perinatal Factors (Hazard Ratios and 95% CIs) **eTable 11. **Risk of Cancer Diagnosed up to 5 Years Among Children Born With Different Perinatal Factors 1973 to 2014 in Sweden (Hazard Ratios and 95% CIs)

Supplement 2.
Data Sharing Statement
