# Supplementary material for: Neurocutaneous Syndromes, Perinatal Factors, and the Risk of Childhood Cancer in Sweden
Source: JAMA Netw Open. 2023 Jul 25;6(7):e2325482. doi: 10.1001/jamanetworkopen.2023.25482 (PMC10370257; doi:10.1001/jamanetworkopen.2023.25482)
Supplement: Supplement 1. — eFigure 1. Cohort Flow Chart eFigure 2. Directed Acyclic Graph of the Study eFigure 3. Cumulative Incidence of Cancer Among Children With and Without a Neurocutaneous Syndrome Diagnosis eTable 1. Distribution of Exposure and Main Outcomes in the Study Population, According to Completeness of Data on the Main Covariates eTable 2. Risk of Cancer Among Children Born 1973 to 2014 With Neurocutaneous Syndromes in Sweden (Unadjusted Hazard Ratios and 95% CIs) eTable 3. Risk of Malignant CNS Among Children Born 1973 to 2014 With Neurocutaneous Syndromes in Sweden (Hazard Ratios and 95% CIs) eTable 4. Risk of Cancer Among Children Born 1973 to 2014 With Neurocutaneous Syndromes in Sweden, Stratified by Birth Period (Hazard Ratios and 95% CIs) eTable 5. Risk of Cancer Among Children Born 1973 to 2014 With Familial Neurocutaneous Syndromes in Sweden (Hazard Ratios and 95% CIs) eTable 6. Risk of Cancer Among Children Born 1973 to 2014 With Neurocutaneous Syndromes in Sweden (at Least 2 Diagnoses of a Neurocutaneous Syndrome; Hazard Ratios and 95% CIs) eTable 7. Risk of Cancer Among Children Born 1973 to 2014 With Neurocutaneous Syndromes in Sweden, Stratified by Age at Cancer Diagnosis (Ratios and 95% CIs) eTable 8. Risk of Cancer Among Children Born With Different Perinatal Factors 1973 to 2014 in Sweden (Unadjusted Hazard Ratios and 95% CIs) eTable 9. Risk of Cancer Among Children Born With Different Perinatal Factors 1973 to 2014 in Sweden, Stratified by Neurofibromatosis Type 1 Diagnosis (Hazard Ratios and 95% CIs) eTable 10. Risk of Cancer Among Children Born 1973 to 2014 With Different Combinations of Neurofibromatosis Type 1 and Specific Perinatal Factors (Hazard Ratios and 95% CIs) eTable 11. Risk of Cancer Diagnosed up to 5 Years Among Children Born With Different Perinatal Factors 1973 to 2014 in Sweden (Hazard Ratios and 95% CIs) [file jamanetwopen-e2325482-s001.pdf]

Supplemental Online Content

Kampitsi CE, Nordgren A, Mogensen H, Pontén E, Feychting M, Tettamanti G. Neurocutaneous syndromes, perinatal factors, and the risk of childhood cancer in Sweden. *JAMA Netw Open*. 2023;6(7):e2325482. doi:10.1001/jamanetworkopen.2023.25482

eFigure 1. Cohort Flow Chart

eFigure 2. Directed Acyclic Graph of the Study

eFigure 3. Cumulative Incidence of Cancer Among Children With and Without a Neurocutaneous Syndrome Diagnosis

eTable 1. Distribution of Exposure and Main Outcomes in the Study Population, According to Completeness of Data on the Main Covariates

eTable 2. Risk of Cancer Among Children Born 1973 to 2014 With Neurocutaneous Syndromes in Sweden (Unadjusted Hazard Ratios and 95% CIs)

eTable 3. Risk of Malignant CNS Among Children Born 1973 to 2014 With Neurocutaneous Syndromes in Sweden (Hazard Ratios and 95% CIs)

eTable 4. Risk of Cancer Among Children Born 1973 to 2014 With Neurocutaneous Syndromes in Sweden, Stratified by Birth Period (Hazard Ratios and 95% CIs)

eTable 5. Risk of Cancer Among Children Born 1973 to 2014 With Familial Neurocutaneous Syndromes in Sweden (Hazard Ratios and 95% CIs)

eTable 6. Risk of Cancer Among Children Born 1973 to 2014 With Neurocutaneous Syndromes in Sweden (at Least 2 Diagnoses of a Neurocutaneous Syndrome; Hazard Ratios and 95% CIs)

eTable 7. Risk of Cancer Among Children Born 1973 to 2014 With Neurocutaneous Syndromes in Sweden, Stratified by Age at Cancer Diagnosis (Ratios and 95% CIs)

eTable 8. Risk of Cancer Among Children Born With Different Perinatal Factors 1973 to 2014 in Sweden (Unadjusted Hazard Ratios and 95% CIs)

eTable 9. Risk of Cancer Among Children Born With Different Perinatal Factors 1973 to 2014 in Sweden, Stratified by Neurofibromatosis Type 1 Diagnosis (Hazard Ratios and 95% CIs)

eTable 10. Risk of Cancer Among Children Born 1973 to 2014 With Different Combinations of Neurofibromatosis Type 1 and Specific Perinatal Factors (Hazard Ratios and 95% CIs)

eTable 11. Risk of Cancer Diagnosed up to 5 Years Among Children Born With Different Perinatal Factors 1973 to 2014 in Sweden (Hazard Ratios and 95% CIs)

This supplemental material has been provided by the authors to give readers additional information about their work.

© 2023 Kampitsi CE et al. *JAMA Network Open.*

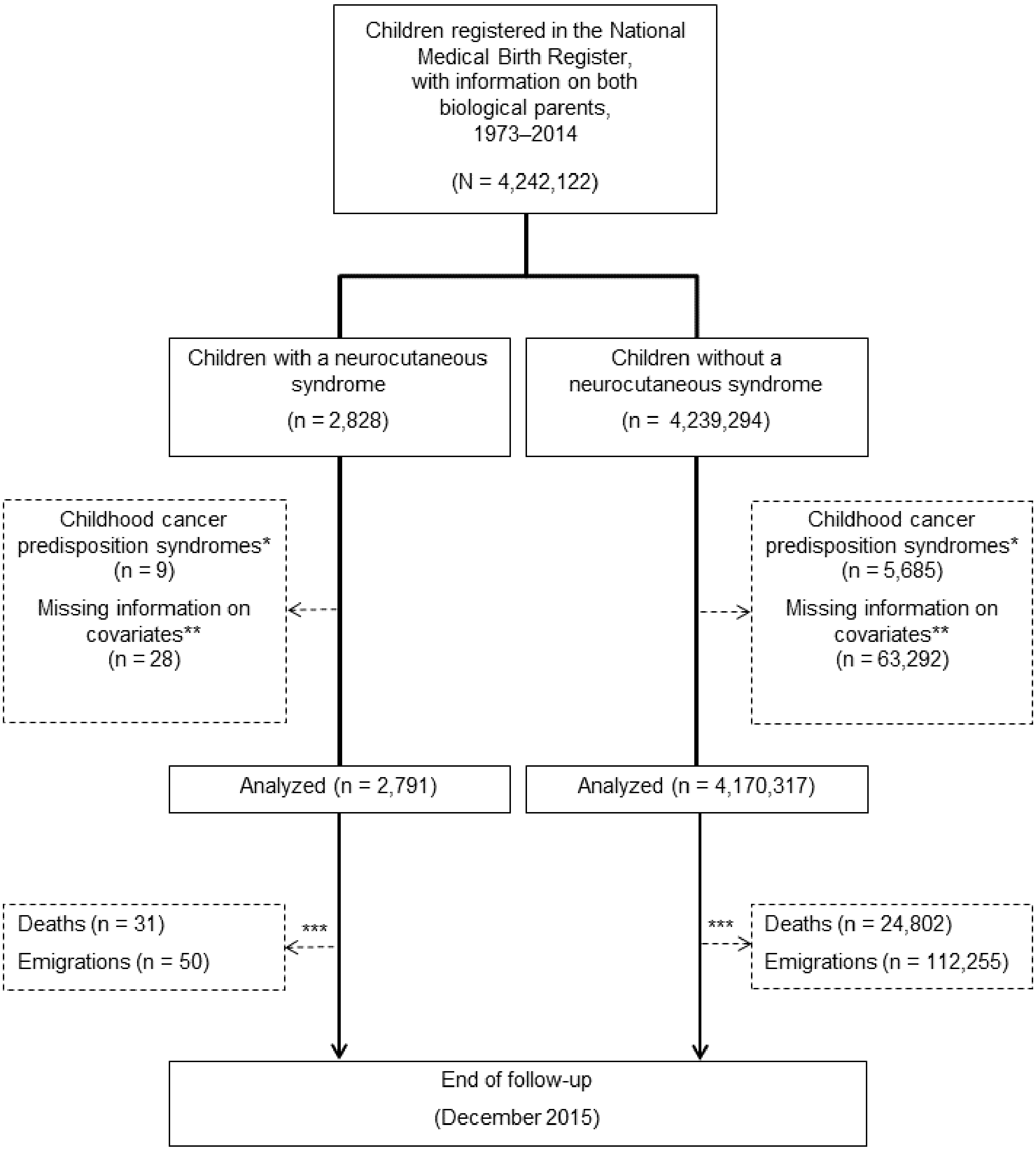

eFigure 1. Cohort flow chart.
* Down syndrome, Beckwith-Wiedemann syndrome, Sotos syndrome, Simpson–Golabi–Behmel syndrome, Perlman syndrome
** Sex, birth decade, region of residence at birth, maternal/paternal education. All children had complete information on birth decade and region of residence at birth.
*** Prior to any cancer diagnosis.

eFigure 1. Cohort Flow Chart

© 2023 Kampitsi CE et al. *JAMA Network Open.*

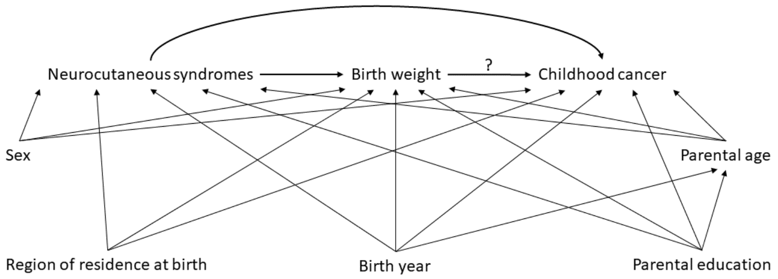

eFigure 2. Directed Acyclic Graph of the Study

© 2023 Kampitsi CE et al. *JAMA Network Open.*

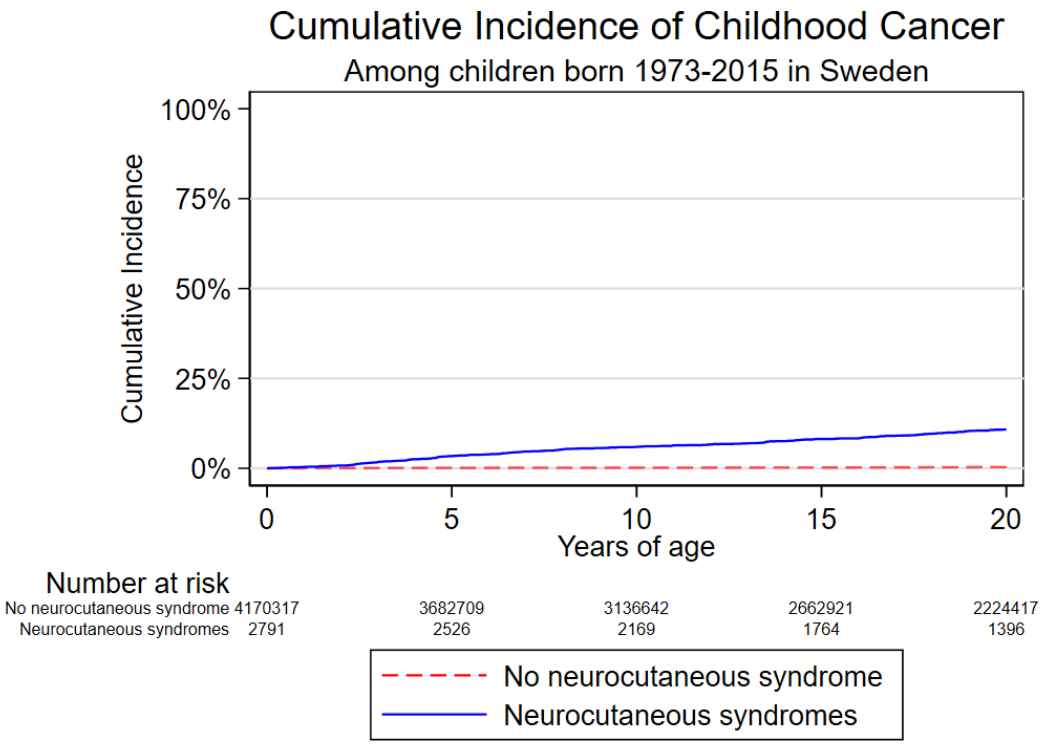

eFigure 3. Cumulative Incidence of Cancer Among Children With and Without a Neurocutaneous Syndrome Diagnosis

© 2023 Kampitsi CE et al. *JAMA Network Open.*

eTable 1. Distribution of Exposure and Main Outcomes in the Study Population, According to Completeness of Data on the Main Covariates

	**Individuals with complete information on covariates (N= 4,173,108; 98.5%)**	**Individuals with missing values on covariates (N=63,320; 1.5%)**
	**n (%)**	**n (%)**
**Neurocutaneous syndromes**		
No neurocutaneous syndrome	4,170,317 (99.93)	63,292 (99.96)
Neurocutaneous syndrome	2,791 (0.07)	28 (0.04)
**Childhood cancer**	10,759 (0.26)	79 (0.12)
CNS	2,896 (0.07)	23 (0.04)
Leukemia	2,849 (0.07)	22 (0.04)
Lymphoma	1,273 (0.03)	6 (<0.01)

Abbreviations: CNS , central nervous system.

© 2023 Kampitsi CE et al. *JAMA Network Open.*

eTable 2. Risk of Cancer Among Children Born 1973 to 2014 With Neurocutaneous Syndromes in Sweden (Unadjusted Hazard Ratios and 95% CIs)

	NS		NF1		TSC		VHL[a]		AT	
	**No. cases NS/no NS**	**HR (95% CI)**	**No. cases NF1/no NF1**	**HR (95% CI)**	**No. cases TSC/no TSC**	**HR (95% CI)**	**No. cases VHL/no VHL**	**HR (95% CI)**	**No. cases AT/no AT**	**HR (95% CI)**
**All cancers**	247/10,512	35.1 (31.0–39.8)	142/10,617	31.3 (26.6–37.0)	36/10,723	31.3 (22.6–43.4)	4/10,575	22.1 (8.3–59.0)	3/10,756	27.5 (8.9–85.3)
**CNS**	203/2,693	111.9 (97.1–129.1)	112/2,784	93.8 (77.7–113.4)	31/2,865	100.3 (70.4–142.9)	<3/2,837	41.0 (10.3–164.1)	NA	NA
**Leukemia**	5/2,844	2.6 (1.1–6.2)	5/2,844	4.1 (1.7–9.8)	NA	NA	NA	NA	NA	NA
**Lymphoma**	4/1,269	4.8 (1.8–12.7)	NA	NA	NA	NA	NA	NA	3/1,270	233.7 (75.3–725.6)

[a] Excluding children who were both born and died between 1987–1996

Abbreviations: NS , Neurocutaneous syndromes ; NF1 , neurofibromatosis 1 ; TSC , tuberous sclerosis complex ; VHL , von Hippel-Lindau; AT , ataxia-telangiectasia ; HR , hazard ratio ; CI , confidence interval ; CNS , central nervous system.

© 2023 Kampitsi CE et al. *JAMA Network Open.*

**eTable 3. Risk of Malignant CNS Among Children Born 1973 to 2014 With Neurocutaneous Syndromes in Sweden (Hazard Ratios and 95% CIs)**		
	**No. CNS cases**	**HR (95% CI)**
**NS**	109/1,636	98.3 (81.0–119.4)
**NF1**	67/1,678	92.5 (72.4–118.1)
**TSC**	24/1,721	127.6 (85.3–190.9)
**VHL**[a]	NA	NA
**AT**	NA	NA

1992 or earlier: Tumors were considered malignant if the third digit of the histopathology code was "6". 1993 or later: Tumors were considered malignant if the fifth digit in the morphology code was "3".

Adjusted for sex, birth decade, maternal/paternal age, maternal/paternal education, region of residence at birth

[a] Excluding children who were both born and died between 1987–1996

Abbreviations: NS , Neurocutaneous syndromes ; NF1 , neurofibromatosis 1 ; TSC , tuberous sclerosis complex ; VHL , von Hippel-Lindau; AT , ataxia-telangiectasia ; HR , hazard ratio ; CI , confidence interval ; CNS , central nervous system.

© 2023 Kampitsi CE et al. *JAMA Network Open.*

eTable 4. Risk of Cancer Among Children Born 1973 to 2014 With Neurocutaneous Syndromes in Sweden, Stratified by Birth Period (Hazard Ratios and 95% CIs)

	NS		NF1		TSC		VHL[a]		AT	
	**No. cases NS/no NS**	**HR (95% CI)**	**No. cases NF1/no NF1**	**HR (95% CI)**	**No. cases TSC/no TSC**	**HR (95% CI)**	**No. cases VHL/no VHL**	**HR (95% CI)**	**No. cases AT/no AT**	**HR (95% CI)**
Born 1973–1995										
**All cancers**	177/7,395	35.9 (30.9–41.6)	100/7,472	31.9 (26.2–38.8)	21/7,551	26.4 (17.2–40.5)	4/7,388	27.0 (10.1–72.1)	3/7,569	37.1 (12.0–115.1)
**CNS**	145/1,948	111.7 (94.3–132.3)	81/2,012	95.9 (76.8–119.7)	16/2,077	73.2 (44.8–119.8)	<3/2,034	49.3 (12.3–197.1)	NA	NA
**Leukemia**	3/1,844	2.4 (0.8–7.4)	3/1,844	3.8 (1.2–11.8)	NA	NA	NA	NA	NA	NA
**Lymphoma**	3/1,030	4.4 (1.4–13.7)	NA	NA	NA	NA	NA	NA	3/1,030	288.9 (92.9–898.4)
Born 1996–2014										
**All cancers**	70/3,117	32.9 (25.9–41.7)	42/3,145	30.0 (22.1–40.6)	15/3,172	41.2 (24.8–68.4)	NA	NA	NA	NA
**CNS**	58/745	113.1 (86.5–147.8)	31/772	89.2 (62.3–127.8)	15/788	162.9 (97.7–271.7)	NA	NA	NA	NA
**Leukemia**	<3/1,000	3.0 (0.7–12.0)	<3/1,000	4.6 (1.1–18.4)	NA	NA	NA	NA	NA	NA
**Lymphoma**	<3/239	5.8 (0.8–41.1)	NA	NA	NA	NA	NA	NA	NA	NA

Adjusted for sex, birth decade, maternal/paternal age, maternal/paternal education, region of residence at birth
[a] Excluding children who were both born and died between 1987–1996

Abbreviations: NS , Neurocutaneous syndromes ; NF1 , neurofibromatosis 1 ; TSC , tuberous sclerosis complex ; VHL , von Hippel-Lindau; AT , ataxia-telangiectasia ; HR , hazard ratio ; CI , confidence interval ; CNS , central nervous system.

© 2023 Kampitsi CE et al. *JAMA Network Open.*

eTable 5. Risk of Cancer Among Children Born 1973 to 2014 With Familial Neurocutaneous Syndromes in Sweden (Hazard Ratios and 95% CIs)

	NS		NF1		TSC		VHL[a]		AT	
	**No. cases NS/no NS**	**HR (95% CI)**	**No. cases NF1/no NF1**	**HR (95% CI)**	**No. cases TSC/no TSC**	**HR (95% CI)**	**No. cases VHL/no VHL**	**HR (95% CI)**	**No. cases AT/no AT**	**HR (95% CI)**
**All cancers**	51/10,708	33.1 (25.1–43.6)	32/10,727	26.2 (18.5–37.1)	5/10,754	44.9 (18.7–107.9)	<3/10,578	12.6 (1.8–89.8)	<3/10,757	62.1 (15.5–248.5)
**CNS**	41/2,855	101.1 (74.3–137.7)	25/2,871	77.7 (52.4–115.2)	5/2,891	170.7 (71.0–410.6)	<3/2,838	47.6 (6.7–338.3)	NA	NA
**Leukemia**	<3/2,848	2.3 (0.3–16.7)	<3/2,848	3.0 (0.4–21.1)	NA	NA	NA	NA	NA	NA
**Lymphoma**	<3/1,270	17.3 (5.6–53.8)	NA	NA	NA	NA	NA	NA	<3/1,271	627.0 (156.2–2518.0

Adjusted for sex, birth decade, maternal/paternal age, maternal/paternal education, region of residence at birth

[a] Excluding children who were both born and died between 1987–1996

Abbreviations: NS , Neurocutaneous syndromes ; NF1 , neurofibromatosis 1 ; TSC , tuberous sclerosis complex ; VHL , von Hippel-Lindau; AT , ataxia-telangiectasia ; HR , hazard ratio ; CI , confidence interval ; CNS , central nervous system.

© 2023 Kampitsi CE et al. *JAMA Network Open.*

eTable 6. Risk of Cancer Among Children Born 1973 to 2014 With Neurocutaneous Syndromes in Sweden (at Least 2 Diagnoses of a Neurocutaneous Syndrome; Hazard Ratios and 95% CIs)

	NS		NF1		TSC		VHL[a]		AT	
	**No. cases NS/no NS**	**HR (95% CI)**	**No. cases NF1/no NF1**	**HR (95% CI)**	**No. cases TSC/no TSC**	**HR (95% CI)**	**No. cases VHL/no VHL**	**HR (95% CI)**	**No. cases AT/no AT**	**HR (95% CI)**
**All cancers**	221/10,538	39.3 (34.4–44.9)	129/10,630	34.1 (28.6–40.6)	32/10,727	35.4 (25.0–50.1)	4/10,575	25.9 (9.7–69.0)	3/10,756	36.2 (11.7–112.2)
**CNS**	183/2,713	126.1 (108.6–146.5)	102/2,794	102.7 (84.2–125.1)	30/2,866	123.3 (86.1–176.7)	<3/2,837	47.6 (11.9–190.4)	NA	NA
**Leukemia**	4/2,845	2.6 (1.0–6.9)	4/2,845	3.9 (1.5–10.4)	NA	NA	NA	NA	NA	NA
**Lymphoma**	4/1,269	6.0 (2.2–16.0)	NA	NA	NA	NA	NA	NA	3/1,270	323.0 (103.9–1003.7)

Adjusted for sex, birth decade, maternal/paternal age, maternal/paternal education, region of residence at birth

[a] Excluding children who were both born and died between 1987–1996

Abbreviations: NS , Neurocutaneous syndromes ; NF1 , neurofibromatosis 1 ; TSC , tuberous sclerosis complex ; VHL , von Hippel-Lindau; AT , ataxia-telangiectasia ; HR , hazard ratio ; CI , confidence interval ; CNS , central nervous system.

© 2023 Kampitsi CE et al. *JAMA Network Open.*

eTable 7. Risk of Cancer Among Children Born 1973 to 2014 With Neurocutaneous Syndromes in Sweden, Stratified by Age at Cancer Diagnosis (Ratios and 95% CIs)

	NS		NF1		TSC		VHL[a]		AT	
	**No. cases NS/no NS**	**HR (95% CI)**	**No. cases NF1/no NF1**	**HR (95% CI)**	**No. cases TSC/no TSC**	**HR (95% CI)**	**No. cases VHL/no VHL**	**HR (95% CI)**	**No. cases AT/no AT**	**HR (95% CI)**
Ages 0-4										
**All cancers**	91/4,169	32.3 (26.2–39.7)	58/4,202	32.0 (24.7–41.5)	9/4,251	19.5 (10.1–37.5)	NA	NA	NA	NA
**CNS**	73/932	114.3 (89.9–145.3)	44/961	103.8 (76.5–141.0)	8/997	73.9 (36.8–148.1)	NA	NA	NA	NA
**Leukemia**	5/1,513	4.9 (2.0–11.8)	5/1,513	7.7 (3.2–18.5)	NA	NA	NA	NA	NA	NA
Ages 5-9										
**All cancers**	63 /2,074	44.9 (35.0–57.6)	40/2,097	44.8 (32.9–61.0)	8/2,129	33.8 (16.9–67.6)	NA	NA	NA	NA
**CNS**	60/704	127.5 (98.2–165.7)	37/727	121.4 (87.6–168.2)	8/756	95.1 (47.4–190.9)	NA	NA	NA	NA
Ages 10-14										
**All cancers**	46/1,847	37.1 (27.7–49.7)	23/1,870	28.9 (19.1–43.6)	10/1,883	49.3 (26.5–91.7)	3/1,881	82.8 (26.7–256.9)	<3/1,892	48.0 (6.8–340.7)
**CNS**	38/573	100.2 (72.1–139.1)	19/592	76.4 (48.3–120.7)	9/602	140.3 (72.6–271.0)	<3/605	169.0 (42.1–677.9)	NA	NA
**Lymphoma**	<3/303	9.5 (2.4–38.3)	NA	NA	NA	NA	NA	NA	<3/304	306.1 (42.9–2183.3)
Ages 15-20										
**All cancers**	47/2,501	29.0 (21.7–38.8)	21/2,527	19.7 (12.7–30.6)	9/2,539	34.6 (18.0–66.7)	<3/2,529	21.7 (3.1–153.9)	<3/2,546	85.5 (21.3–342.3)
**CNS**	32/507	97.8 (68.4–139.8)	12/527	55.3 (31.2–98.0)	6/533	108.8 (48.6–243.3)	NA	NA	NA	NA
**Lymphoma**	<3/536	5.9 (1.5–23.7)	NA	NA	NA	NA	NA	NA	<3/536	412.1 (102.6–1655.0)

[a] Excluding children who were both born and died between 1987–1996

Abbreviations: NS , Neurocutaneous syndromes ; NF1 , neurofibromatosis 1 ; TSC , tuberous sclerosis complex ; VHL , von Hippel-Lindau; AT , ataxia-telangiectasia ; HR , hazard ratio ; CI , confidence interval ; CNS , central nervous system.

No lymphomas were diagnosed between ages 0-9 among children with any neurocutaneous syndrome.
No leukemias were diagnosed between ages 5-20 among children with any neurocutaneous syndrome.

© 2023 Kampitsi CE et al. *JAMA Network Open.*

eTable 8. Risk of Cancer Among Children Born With Different Perinatal Factors 1973 to 2014 in Sweden (Unadjusted Hazard Ratios and 95% CIs)

	All cancers (n=10,759)		CNS (n=2,896)		Leukemia (n=2,849)		Lymphoma (n=1,273)	
	**No.cases**	**HR (95% CI)**	**No.cases**	**HR (95% CI)**	**No.cases**	**HR (95% CI)**	**No.cases**	**HR (95% CI)**
**Birth weight category**								
Low	448	1.1 (1.0–1.2)	115	1.0 (0.8–1.2)	121	1.1 (0.9–1.3)	47	1.0 (0.7–1.3)
Normal	8,167	1 [Reference]	2,233	1 [Reference]	2,141	1 [Reference]	949	1 [Reference]
High	2,101	1.2 (1.1–1.2)	531	1.1 (1.0–1.2)	577	1.2 (1.1–1.3)	272	1.3 (1.1–1.5)
**Birth weight for gestational age**								
SGA	1,038	1.0 (0.9–1.1)	277	1.0 (0.8–1.1)	291	1.0 (0.9–1.2)	123	1.0 (0.8–1.2)
AGA	8,401	1 [Reference]	2,285	1 [Reference]	2,220	1 [Reference]	979	1 [Reference]
LGA	1,289	1.2 (1.1–1.3)	323	1.1 (1.0–1.2)	334	1.2 (1.0–1.3)	166	1.3 (1.1–1.6)
**5-min Apgar score**								
0–3	31	1.4 (1.0–1.9)	7	1.2 (0.5–2.4)	5	0.8 (0.3–2.0)	3	1.0 (0.3–3.3)
4–6	98	1.2 (1.0–1.5)	24	1.1 (0.8–1.7)	26	1.2 (0.8–1.8)	<3	0.2 (0.1–0.8)
7–10	9,867	1 [Reference]	2,627	1 [Reference]	2,629	1 [Reference]	1,191	1 [Reference]
**Head circumference**								
<33 cm	804	1.1 (1.0–1.2)	202	0.9 (0.8–1.1)	212	1.1 (1.0–1.3)	106	1.3 (1.0–1.6)
33–34 cm	3,458	1 [Reference]	1,023	1 [Reference]	867	1 [Reference]	375	1 [Reference]
35 cm	2,640	1.0 (1.0–1.1)	677	0.9 (0.8–1.0)	745	1.2 (1.1–1.3)	303	1.1 (1.0–1.3)
36–37 cm	3,070	1.1 (1.1–1.2)	790	1.0 (0.9–1.1)	828	1.2 (1.1–1.3)	407	1.4 (1.2–1.6)
>37 cm	415	1.3 (1.2–1.5)	106	1.1 (0.9–1.4)	110	1.4 (1.1–1.6)	50	1.5 (1.1–2.1)
**Preterm birth**	649	1.1 (1.0–1.2)	168	1.1 (0.9–1.2)	163	1.0 (0.8–1.2)	69	1.0 (0.8–1.3)

Abbreviations: HR , hazard ratio ; CI , confidence interval ; CNS , central nervous system; SGA , small for gestational age ; AGA , appropriate for gestational age ; LGA , large for gestational age.

© 2023 Kampitsi CE et al. *JAMA Network Open.*

eTable 9. Risk of Cancer Among Children Born With Different Perinatal Factors 1973 to 2014 in Sweden, Stratified by Neurofibromatosis Type 1 Diagnosis (Hazard Ratios and 95% CIs)

	All cancers		CNS		Leukemia	
	**No.cases**	**HR (95% CI)**	**No.cases**	**HR (95% CI)**	**No.cases**	**HR (95% CI)**
**Children without NF1**						
**Birth weight category**						
Low	442	1.1 (1.0–1.2)	109	1.0 (0.8–1.2)	121	1.1 (0.9–1.3)
Normal	8,065	1 [Reference]	2,153	1 [Reference]	2,138	1 [Reference]
High	2,068	1.1 (1.1–1.2)	506	1.1 (1.0–1.2)	575	1.2 (1.1–1.3)
**Birth weight for gestational age**						
SGA	1,026	1.0 (0.9–1.0)	267	1.0 (0.8–1.1)	290	1.0 (0.9–1.2)
AGA	8,303	1 [Reference]	2,207	1 [Reference]	2,219	1 [Reference]
LGA	1,257	1.2 (1.1–1.2)	299	1.1 (0.9–1.2)	331	1.2 (1.0–1.3)
**5-min Apgar score**						
0–3	31	1.4 (1.0–2.0)	7	1.2 (0.5–2.5)	5	0.8 (0.3–2.0)
4–6	94	1.2 (1.0–1.5)	21	1.0 (0.7–1.6)	26	1.2 (0.8–1.8)
7–10	9,740	1 [Reference]	2,528	1 [Reference]	2,624	1 [Reference]
**Head circumference**						
<33 cm	800	1.1 (1.0–1.2)	198	0.9 (0.8–1.1)	212	1.1 (1.0–1.3)
33–34 cm	3,406	1 [Reference]	981	1 [Reference]	865	1 [Reference]
35 cm	2,612	1.0 (1.0–1.1)	656	0.9 (0.8–1.0)	744	1.2 (1.0–1.3)
36–37 cm	3,025	1.1 (1.0–1.2)	755	1.0 (0.9–1.1)	827	1.2 (1.0–1.3)
>37 cm	407	1.3 (1.1–1.4)	100	1.1 (0.9–1.4)	109	1.3 (1.0–1.6)
**Preterm birth**	635	1.1 (1.0–1.2)	155	1.0 (0.9–1.2)	163	1.0 (0.9–1.2)
**Children with NF1**						
**Birth weight category**						
Low	6	1.1 (0.4–2.3)	6	1.3 (0.6–3.0)	NA	NA
Normal	102	1 [Reference]	80	1 [Reference]	3	1 [Reference]
High	33	1.2 (0.8–1.8)	25	1.2 (0.8–1.9)	<3	2.9 (0.5–20.3)
**Birth weight for gestational age**						
SGA	12	1.1 (0.6–2.0)	10	1.1 (0.7–2.2)	<3	5.7 (0.4–96.2)
AGA	98	1 [Reference]	78	1 [Reference]	<3	1 [Reference]
LGA	32	1.5 (1.1–2.3)	24	1.5 (1.0–2.3)	3	11.7 (1.2–118.8)
**5-min Apgar score**						
0–3	NA	NA	NA	NA	NA	NA
4–6	4	2.9 (1.1–7.9)	3	2.8 (0.9–9.1)	NA	NA
7–10	127	1 [Reference]	99	1 [Reference]	5	1 [Reference]
**Head circumference**						
<33 cm	4	0.3 (0.1–1.0)	4	0.5 (0.2–1.1)	NA	NA
33–34 cm	52	1 [Reference]	42	1 [Reference]	<3	1 [Reference]
35 cm	28	0.8 (0.5–1.2)	21	0.7 (0.5–1.2)	<3	0.6 (0.1–8.3)
36–37 cm	45	1.1 (0.7–1.5)	35	1.0 (0.7–1.6)	<3	0.5 (0.1–6.0)
>37 cm	8	1.4 (0.7–3.0)	6	1.4 (0.6–3.2)	<3	9.8 (0.6–169.1)
**Preterm birth**	14	1.3 (0.8–2.2)	13	1.5 (0.9–2.7)	NA	NA

Abbreviations:
HR , hazard ratio ; CI , confidence interval ; CNS , central nervous system; NF1 , neurofibromatosis type 1 ; SGA , small for gestational age ; AGA , appropriate for gestational age ; LGA , large for gestational age.

© 2023 Kampitsi CE et al. *JAMA Network Open.*

eTable 10. Risk of Cancer Among Children Born 1973 to 2014 With Different Combinations of Neurofibromatosis Type 1 and Specific Perinatal Factors (Hazard Ratios and 95% CIs)

	All cancers		Leukemia		CNS	
	**No. cases**	**HR (95% CI)**	**No. cases**	**HR (95% CI)**	**No. cases**	**HR (95% CI)**
**Preterm birth**						
no NF1, no preterm	9,982	1 [Reference]	2,681	1 [Reference]	2,629	1 [Reference]
no NF1, preterm	635	1.1 (1.0–1.2)	163	1.1 (0.9–1.2)	155	1.0 (0.9–1.2)
NF1, no preterm	128	30.8 (25.9–36.7)	5	4.4 (1.8–10.7)	99	90.3 (73.9–110.4)
NF1, preterm	14	38.2 (22.6–64.4)[a]	NA	NA	13	133.7 (77.5–230.6)[a]
**Large head circumference**						
no NF1, no large head	10,210	1 [Reference]	2,735	1 [Reference]	2,684	1 [Reference]
no NF1, large head	407	1.2 (1.1–1.3)	109	1.2 (1.0–1.4)	100	1.1 (0.9–1.4)
NF1, no large head	134	30.8 (26.0–36.6)	4	3.4 (1.3–9.1)	106	92.5 (76.2–112.4)
NF1, large head	8	48.0 (23.8–95.1)[a]	<3	20.6 (2.9–146.5)[a]	6	139.0 (62.4–309.8)[a]
**LGA**						
no NF1, no LGA	9,360	1 [Reference]	2,513	1 [Reference]	2,485	1 [Reference]
no NF1, LGA	1,257	1.2 (1.1–1.2)	331	1.2 (1.0–1.3)	299	1.1 (0.9–1.2)
NF1, no LGA	110	29.4 (24.4–35.5)	<3	2.0 (0.5–7.9)	88	88.5 (71.5–109.5)
NF1, LGA	32	44.1 (31.2–62.4)[a]	3	15.1 (4.9–46.8)[b]	24	124.4 (83.2–186.0)[a]

Adjusted for sex, birth decade, maternal/paternal age, maternal/paternal education, region of residence at birth

Abbreviations:
NF1 , neurofibromatosis 1 ; CNS , central nervous system; HR , hazard ratio ; CI , confidence interval ; LGA , large for gestational age.

[a] p-value for interaction>0.05
[b] p-value for interaction<0.05

© 2023 Kampitsi CE et al. *JAMA Network Open.*

eTable 11. Risk of Cancer Diagnosed up to 5 Years Among Children Born With Different Perinatal Factors 1973 to 2014 in Sweden (Hazard Ratios and 95% CIs)

	**All cancers**		**CNS**		**Leukemia**	
**Birth weight category**	**No. cases**	**HR (95% CI)**	**No. cases**	**HR (95% CI)**	**No. cases**	**HR (95% CI)**
Low	448	1.0 (0.9–1.2)	115	1.1 (0.8–1.5)	121	1.0 (0.6–1.3)
Normal	8,167	1 [Reference]	2,233	1 [Reference]	2,141	1 [Reference]
High	2,101	1.1 (1.1–1.2)	531	1.1 (1.0–1.3)	577	1.2 (1.0–1.3)
**Birth weight for gestational age**						
SGA	1,038	0.9 (0.8–1.0)	277	0.9 (0.7–1.1)	291	0.9 (0.8–1.1)
AGA	8,401	1 [Reference]	2,285	1 [Reference]	2,220	1 [Reference]
LGA	1,289	1.2 (1.0–1.3)	323	1.0 (0.8–1.2)	334	1.2 (1.0–1.4)
**5-min Apgar score**						
0–3	31	2.0 (1.2–3.2)	7	1.5 (0.5–4.5)	5	0.6 (0.2–2.6)
4–6	98	1.5 (1.1–1.9)	24	1.3 (0.7–2.5)	26	1.5 (1.0–2.3)
7-10	9,867	1 [Reference]	2,627	1 [Reference]	2,629	1 [Reference]
**Head circumference**						
<33 cm	804	1.0 (0.9–1.2)	202	0.9 (0.7–1.1)	212	1.2 (1.0–1.4)
33–34 cm	3,458	1 [Reference]	1,023	1 [Reference]	867	1 [Reference]
35 cm	2,640	1.1 (1.0–1.2)	677	0.9 (0.8–1.1)	745	1.2 (1.1–1.4)
36–37 cm	3,070	1.1 (1.0–1.2)	790	1.0 (0.9–1.2)	828	1.2 (1.1–1.4)
>37 cm	415	1.4 (1.2–1.6)	106	1.2 (0.9–1.7)	110	1.5 (1.1–1.9)
**Preterm birth**	649	1.2 (1.1–1.3)	168	1.2 (0.9–1.5)	163	1.2 (1.0–1.6)

Adjusted for sex, birth decade, maternal/paternal age, maternal/paternal education, region of residence at birth

Abbreviations:
HR , hazard ratio ; CI , confidence interval ; CNS , central nervous system; SGA , small for gestational age ; AGA , appropriate for gestational age ; LGA , large for gestational age.

© 2023 Kampitsi CE et al. *JAMA Network Open.*
