# Supplementary material for: Neurocutaneous Syndromes, Perinatal Factors, and the Risk of Childhood Cancer in Sweden
Source: JAMA Netw Open. 2023 Jul 25;6(7):e2325482. doi: 10.1001/jamanetworkopen.2023.25482 (PMC10370257; doi:10.1001/jamanetworkopen.2023.25482)
Supplement: Supplement 2. — Data Sharing Statement [file jamanetwopen-e2325482-s002.pdf]

Data Sharing Statement

Kampitsi. Neurocutaneous Syndromes, Perinatal Factors, and the Risk of Childhood Cancer in Sweden. *JAMA Netw Open*. Published July 25, 2023.
doi:10.1001/jamanetworkopen.2023.25482

Data
Data available: No

Additional Information
Explanation for why data not available: Swedish laws and regulations do not allow sharing of personal sensitive data, which can only be made available for researchers who fulfill legal requirements for access to personal sensitive data. Eligible individuals can apply for the data from the National Board of Health and Welfare in Sweden (https://www.socialstyrelsen.se/statistik-och-data/bestalla-data-och-statistik/) and from Statistics Sweden (https://www.scb.se/vara-tjanster/bestall-data-och-statistik/).
